# Therapeutic implications of an enriched cancer stem-like cell population in a human osteosarcoma cell line

**DOI:** 10.1186/1471-2407-12-139

**Published:** 2012-04-04

**Authors:** Sara R Martins-Neves, Áurio O Lopes, Anália do Carmo, Artur A Paiva, Paulo C Simões, Antero J Abrunhosa, Célia MF Gomes

**Affiliations:** 1 Pharmacology and Experimental Therapeutics - Institute of Biomedical Research in Light and Image (IBILI), Faculty of Medicine, University of Coimbra, Az. de Sta. Comba, Celas, Coimbra 3000-354, Portugal; 2 Center for Neurosciences and Cell Biology (CNC), Coimbra, Portugal; 3 Center of Investigation in Environment, Genetics and Oncobiology (CIMAGO), Faculty of Medicine, University of Coimbra, Coimbra, Portugal; 4 Histocompatibility Centre, University Hospital of Coimbra, Coimbra, Portugal; 5 Radiotherapy Service, University Hospital of Coimbra, Coimbra, Portugal; 6 Institute for Nuclear Sciences Applied to Health (ICNAS), University of Coimbra, Coimbra, Portugal

**Keywords:** Osteosarcoma, Cancer stem-like cells, Resistance, Chemotherapy, Radiotherapy

## Abstract

**Background:**

Osteosarcoma is a bone-forming tumor of mesenchymal origin that presents a clinical pattern that is consistent with the cancer stem cell model. Cells with stem-like properties (CSCs) have been identified in several tumors and hypothesized as the responsible for the relative resistance to therapy and tumor relapses. In this study, we aimed to identify and characterize CSCs populations in a human osteosarcoma cell line and to explore their role in the responsiveness to conventional therapies.

**Methods:**

CSCs were isolated from the human MNNG/HOS cell line using the sphere formation assay and characterized in terms of self-renewal, mesenchymal stem cell properties, expression of pluripotency markers and ABC transporters, metabolic activity and tumorigenicity. Cell's sensitivity to conventional chemotherapeutic agents and to irradiation was analyzed and related with cell cycle-induced alterations and apoptosis.

**Results:**

The isolated CSCs were found to possess self-renewal and multipotential differentiation capabilities, express markers of pluripotent embryonic stem cells Oct4 and Nanog and the ABC transporters P-glycoprotein and BCRP, exhibit low metabolic activity and induce tumors in athymic mice. Compared with parental MNNG/HOS cells, CSCs were relatively more resistant to both chemotherapy and irradiation. None of the treatments have induced significant cell-cycle alterations and apoptosis in CSCs.

**Conclusions:**

MNNG/HOS osteosarcoma cells contain a stem-like cell population relatively resistant to conventional chemotherapeutic agents and irradiation. This resistant phenotype appears to be related with some stem features, namely the high expression of the drug efflux transporters P-glycoprotein and BCRP and their quiescent nature, which may provide a biological basis for resistance to therapy and recurrence commonly observed in osteosarcoma.

## Background

Osteosarcoma is the most common malignant primary bone tumor comprising 20% of all bone tumors and about 5% of pediatric tumors overall [1]. Significant improvements in patient survival rates have been achieved in recent years, largely due to multimodal therapeutic approaches combining high-dose chemotherapy and surgical resection [2]. Radiotherapy, although not a primary choice for treatment, is frequently applied for local control in patients for whom surgical resection with sufficient margins is not achievable [3,4]. Despite these advances, the overall relapse free-survival rate over 5-years has stagnated at approximately 65% to 75% and the intensification of chemotherapy regimens has improved histological response but not survival [5,6].

There is increasing evidence that tumors are hierarchically organized and sustained by a subset of cells with attributes of stem cells that are refractory to conventional therapies [7]. These cells, referred to as cancer stem-like cells (CSCs) or, alternatively, tumor-initiating cells, share several characteristics with embryonic and somatic stem cells including self-renewal and differentiation abilities, and represent a small fraction of the cellular population of a tumor [8].

Recent reports have found that tumor cells expressing stem markers are able to initiate solid tumors in immunodeficient mice recapitulating the heterogeneity of the original tumors, supporting the theory that residual undifferentiated cells contain the complete genetic programs necessary to initiate tumorigenesis and sustain the growth of the tumor bulk [9]. Studies performed in glioblastoma and breast cancer support the theory that CSCs have innate survival advantages compared with more differentiated cells, allowing them to survive after therapy and regenerate the tumor [10]. This phenotype appears to be related with properties they share with normal stem cells, such as the higher capacity for DNA repair, quiescent status and the overexpression of ATP-binding cassette drug transporters including P-glycoprotein (Pgp) and the breast cancer resistance protein (BCRP) [11,12]. These transmembrane proteins behave as drug efflux pumps of most chemotherapeutic agents preventing their intracellular accumulation at toxic concentrations [13]. Since Pgp and BCRP recognize most conventional chemotherapeutic drugs as transport substrates, it is likely that they contribute largely to a chemotherapy-resistant phenotype when expressed by CSCs [12]. The relative quiescence and slow cycling rate of CSCs render them refractory to therapies that rely on cell cycle kinetics to induce lethal cellular damage in highly proliferative cells [14]. This was observed in leukemia stem cells isolated from acute myelogenous leukemia, which, due to their quiescence, proved to be less sensitive to chemotherapy [15].

Recently, a subpopulation of cancer cells with stem-like properties was identified in bone sarcomas [16,17]. These cells were found to express surface markers of mesenchymal stem cells (MSCs) as well as ability to differentiate along mesenchymal lineages (osteogenic, adipogenic and chondrogenic), which suggest that sarcomas arise from cells at least as primitive as MSCs that undergo oncogenic transformation and contain a subpopulation of cells with attributes of stem cells. The evidence of such cells in bone sarcomas may provide a rational explanation for the recurrence often observed in such aggressive tumors and help us to understand why this tumor is so difficult to eradicate. Although several retrospective studies have identified dose intensity as a potential determinant factor in survival of osteosarcoma patients, the results of the third European Osteosarcoma Intergroup (EOI) randomized controlled trial in osteosarcoma showed that the increment in dose intensity, while improving histologic response, did not translate into a demonstrable patient benefit in overall survival or progression-free survival [6]. Although intensified chemotherapy kills cancer cells and shrinks the tumors, it is likely that a minority of cells escapes treatments and has tumor initiating ability. If these are truly CSCs and given the specific stem cell features, the current therapeutic approaches may not address this subset of cells leading to relapse, which emphasizes the need to develop more effective therapeutic strategies targeting CSCs [18]. Therefore, a better understanding of CSCs' response to conventional therapies is essential to understand the biological consequences of their existence in clinical response and to provide targets for new CSC-directed therapies. In the present study, we aimed to evaluate the role of osteosarcoma cells with stem features in the responsiveness to conventional therapies including the current chemotherapeutic agents recommended by European and American Osteosarcoma group (EURAMOS-1) and to ionizing irradiation.

We have identified a subpopulation of cells with stem-like properties in a human osteosarcoma cell line that is relatively resistant to both conventional chemo- and radiotherapy. This resistant phenotype appears to be related with stem features, namely the high expression of drug transporters and quiescent status. Based on these observations, we believe that CSCs play a critical role in determining the response of osteosarcoma patients to therapy and should be considered when designing new anti-cancer therapies.

## Methods

### Cell culture and sphere-forming assay

Human MNNG/HOS osteosarcoma cell line was purchased from the American Type Culture Collection (Rockville, MD) and was cultured in RPMI 1640 medium (Gibco, Scotland, UK) supplemented with 10% heat inactivated fetal bovine serum (FBS) at 37°C in a humidified incubator with 95% air and 5% CO_2_. The sphere forming assay was performed as described previously by Gibbs *et al. *with minor modifications [16]. After reaching 60-80% confluence, cells were harvested and plated at a density of 60,000 cells/well on 6-well poly-HEMA-coated plates (Sigma, St Louis, USA) in serum-free DMEM/F12 medium (Sigma) with 1% of methylcellulose (Sigma) supplemented with 1% penicillin/streptomycin (Gibco), 20 nM progesterone (Sigma), 100 μM putrescine (Sigma), 1% insulin-transferrin-selenium A supplement (Gibco), 10 ng/ml basic fibroblast growth factor (bFGF, Peprotech EC, London, UK) and 10 ng/ml human recombinant epidermal growth factor (EGF, Sigma). Fresh aliquots of growth factors were added twice a week. After 7 days of culturing, the formed cellular spheres (sarcospheres) were collected, dissociated with accutase and re-seeded again in stressful growth conditions for formation of secondary spheres. This procedure was repeated at least three times to evaluate the self-renewing capacity of the spherical clones in anchorage-independent conditions. The total number of spherical colonies obtained at each passage was quantitated under an inverted phase contrast microscope. The sphere-forming efficiency at each passage was calculated by dividing the number of spheres formed by the total number of cells seeded and expressed as a percentage.

A third generation sphere culture was transferred to adherent plates and allowed to grow in monolayer in culture medium supplemented with 10% FBS without growth factors. This sphere-derived monolayer culture is referred to as SAR-OS cells and was expanded and used in some subsequent studies to verify if these cells derived directly from sarcospheres acquired the same biological behavior as parental MNNG/HOS cells.

### Flow cytometry analysis of mesenchymal stem cell markers

The immunophenotype of sarcospheres was analyzed for the expression of surface antigens associated with MSCs according to the International Society for Cellular Therapy (ISCT) [19]. Cells derived from monolayer cultures and 7-day old sarcospheres were enzymatically dissociated with Trypsin-EDTA and Accutase (Sigma), respectively, and re-suspended in phosphate-buffered saline solution (PBS). Single-cell suspensions (1 × 10^6^/ml) were then incubated with PE-conjugated anti-CD105 (Immunostep), PE-conjugated anti-CD73 (BD Pharmingen), APC-conjugated anti-90 (BD Pharmingen) and PO-conjugated anti-CD13 (BD Pharmingen). Negative control staining was performed with the APCH7-conjugated anti-CD45 (BD Pharmingen), PB-conjugated anti-CD11b (BD Pharmingen), PE-Cy7-conjugated anti-CD19 (eBioscience), PerCP-Cy5.5-conjugated anti-CD34 (BD Pharmingen) and FITC-conjugated anti-HLA-DR (eBioscience). Data were acquired on a FACSCalibur flow cytometer (Beckton-Dickinson, San Diego, CA, USA) and analyzed using the CellQuest software (BD Biosciences).

### Multilineage differentiation studies

MNNG/HOS cells and clonally-derived sarcospheres were induced to differentiate towards mesenchymal lineages (osteoblasts, chondrocytes and adipocytes) using the STEMPRO^® ^differentiation kits (Gibco) according to the manufacturer's instructions. In brief, after expansion in MSC growth medium (low glucose DMEM supplemented with 10% of MSC qualified FBS, 2 mM glutamine and 1% penicillin/streptomycin), cells were seeded into StemPro^® ^Osteogenesis, Adipogenesis or Chondrogenesis Differentiation medium in 12-well plates. The induction medium was changed every 3 to 4 days during the incubation period. Osteogenic differentiation was assessed after 21 days of incubation. Cells were fixed with 4% formaldehyde and stained with 2% Alizarin Red S (Sigma) to identify the formation of calcium deposits. To visualize adipogenic differentiation, cultures were fixed with 4% formaldehyde and stained with Oil Red O (Sigma, 3 mg Oil Red O/ml 60% isopropanol) after 14 days in adipogenic induction medium. Chondrogenic differentiation was monitored after 16 days by staining micromass pellet cultures with 1% Alcian Blue (Sigma) for proteoglycan matrix production. Images were taken using an inverted fluorescence microscope (Nikon, Eclipse TS 100) and Motic Images Advanced 3.2 software.

### Western blot analysis

Cells were lysed in a RIPA buffer containing protease and phosphatase inhibitors (Roche, Germany). Protein concentration was determined using the BCA protein assay kit (Merck, Germany). About 40 μg of total extract protein from MNNG/HOS cells and sarcospheres were separated by SDS-PAGE and then electrotransferred to activated polyvinylidene difluoride membranes. Non-specific protein binding was blocked by incubating the membranes for 1 h with 5% non-fat dry milk in 0.1% TBS-T. Membranes were incubated for 2 h at room temperature with primary antibodies at a dilution of 1:1000 for human monoclonal anti-Oct4 (Cell Signaling), 1:1000 for human monoclonal anti-Nanog (Cell Signaling), 1:150 for human monoclonal anti-Pgp (Calbiochem) and 1:250 for human monoclonal anti-BCRP (Millipore). The membranes were then washed in TBS-T and incubated for 1 h in horseradish peroxidase-conjugated secondary anti-rabbit or anti-mouse antibody at a dilution of 1:20000. The proteins were visualized by chemifluorescence (ECF™ Western Blotting Reagent Pack, GE Lifesciences, Pittsburg, PA) using Typhoon™ FLA 9000 imager. Fold change in protein expression was expressed as a ratio calculated by dividing the specific protein band density by the β-actin band density and then normalized to the control.

### Animal care

Six-week-old male Balb/c nude mice were obtained from Charles River Laboratories and housed under pathogen-free conditions in individual ventilated cages. Sterile food and water were provided *ad libitum*. The animal experiments were performed according to the local and international guidelines on animal experimentation and were approved by the Institutional Ethics Committee of the Faculty of Medicine of University of Coimbra for animal care and use (Approval ID:38-CE-2011, additional file 1).

### Tumorigenic potential of sarcospheres

The tumorigenic potential of sarcospheres was assessed through their ability to generate tumors in immunocompromised mice. One hundred thousand dissociated cells from spherical colonies and the same number of parental cells (MNNG/HOS) were re-suspended in 200 μl of PBS and injected subcutaneously (*s.c*.) into opposite flanks. Tumors' development was monitored weekly for up to 6 weeks and the volume was calculated by V = (length × width^2^)/2. The animals were sacrificed by cervical dislocation when the tumors reached 1.5 cm^3^.

### Metabolic activity

The metabolic activity of sarcospheres and of adherent cells was measured based on cellular uptake of the glucose analogue [^18^F]-2-fluoro-2-deoxy-D-glucose (FDG). FDG was provided by the Institute for Nuclear Sciences Applied to Health (ICNAS), Portugal. Single-cell suspensions (2 × 10^6 ^cells/ml) derived from spherical clones and of monolayer cultures were incubated with 0.75 MBq/ml of FDG at 37°C. At 15, 30 and 60 min samples of 200 μl were taken and transferred to microcentrifuge tubes containing 500 μl of ice-cold PBS and washed twice in PBS. Cell pellets and supernatants were assayed for γ-radioactivity in a well-type sodium iodide γ-counter (SR3, Nuclear Enterprises, Reading, UK) within the ^18^F-sensitivity energy window set as 20%. Results are reported as the percentage of cell-radioactivity associated to the total radioactivity added and normalized per million of cells.

### Drug cytotoxicity assays

We analyzed the chemosensitivity of both sarcospheres and adherent cells (MNNG/HOS and SAR-OS) to the chemotherapeutic agents recommended by the EURAMOS -1 protocol for the treatment of OS that includes doxorubicin (DOX, DOXO-cell^®^, Portugal), cisplatin (CIS, Teva Pharma, Portugal) and methotrexate (MTX, Teva Pharma). Cell viability was analyzed using the 3-(4,5-dimethylthiazol-2-yl)-2.5-diphenyl tetrazolium bromide (MTT, Sigma) assay. Dissociated 7-day old sarcospheres and adherent cells (MNNG/HOS and SAR-OS) were seeded in 24-well plates (25 × 10^3 ^cells/well), allowed to attach overnight and then subsequently treated with increasing concentrations of DOX (0.001 - 60 μM), CIS (0.001 - 80 μM) and MTX (0.001 - 1000 μM). Forty-eight hours after treatment, 200 μl of MTT solution (0.5 mg/ml) was added to each well, and incubation continued for an additional 4 hours. Formed blue formazan crystals were dissolved by adding 200 μL of acidified isopropanol (0.04 N HCl). The solubilized products were transferred to 96 well-plates and the absorbance was read in a microplate reader (Synergy™ HT, Biotek Instruments) at 570 nm using a 620 nm filter as reference. Cytotoxicity was expressed as the percentage of cells surviving in relation to untreated cells. The drug concentration required to inhibit growth by 50% (IC_50_) was estimated with Origin 8.0 (OriginLab Corporation), using the dose-response equation:

$$\text{y}={\text{A}}_{1}+\frac{{\text{A}}_{1}-{\text{A}}_{2}}{1+10^{\left(\text{Log}{\text{x}}_{0}-\text{x}\right)\text{p}}}$$

where A_1 _and A_2 _are the amplitude of the baseline and maximum response, respectively, x_0 _is the IC_50 _and p is the slope.

### Reversal of resistance to DOX

In order to explore whether Pgp and BCRP are functionally active and can account for the higher chemoresistance of sarcospheres, we evaluated the effects of verapamil (VER), a potent inhibitor of Pgp and BCRP on reversal of DOX resistance. DOX is a transport substrate of both Pgp and BCRP. Cells dissociated from sarcospheres and from adherent cells, MNNG/HOS and SAR-OS, were incubated with increasing concentrations of DOX in the presence of non-toxic concentrations of VER (10 μM) during 48 h. The IC_50 _value was calculated as described above.

### Irradiation assay

Single-cell suspensions of sarcospheres and of adherent cultures (MNNG/HOS and SAR-OS) were placed in plastic tubes filled with culture medium and irradiated with single doses of 2, 4, 6, 8, 10, 15 and 20 Gy in a linear accelerator (Varian Clinac 600 C) at a dose rate of 2.70 Gy/min. To assure that cells received a uniform radiation exposure, tubes containing cells were submersed in water in an acrylic container positioned with its long axis parallel to the central axis of the beam. Corresponding controls were sham-irradiated. After irradiation, cells were seeded in 24-well plates at a density of 10 × 10^3 ^cells/well for adherent cells and 50 × 10^3 ^cells for sarcosphere-derived cells and cultured for 7 days. Cell survival was determined using the MTT colorimetric assay. Surviving fractions for each irradiation dose were normalized to the values of sham-irradiated corresponding controls. Cell survival curves were fitted in Origin 8.0, using the linear-quadratic model (LQM), according to the equation:

$$\text{SF}={\text{e}}^{-\alpha \text{D}-\beta {\text{D}}^{2}}$$

in which SF is the surviving fraction at a dose D, α is the log cells killed per Gy of the linear component and is regarded as an estimate of the initial irreparable DNA damage (double-strand breaks, DSB), and β is the log cells killed per Gy^2 ^of the quadratic component and represents the capacity for DNA repair of sub-lethal damage (single-strand breaks, SSB). The median lethal dose (LD_50_) corresponding to the irradiation dose that kills half of the cells was calculated as an estimate of the intrinsic radiosensitivity for each cell line.

### Reactive oxygen species formation assay

The formation of reactive oxygen species (ROS) induced by irradiation was assayed using the fluorescent dye 2'7'-dichlorofluorescein diacetate (H_2_DCF-DA, Gibco) according to the manufacturer instructions. In brief, single-cell suspensions were incubated with 10 μM H_2_DCF-DA for 30 min in the dark. The cells were then washed to remove the loading buffer and allowed to recover in a pre-warmed growth medium. Before irradiation, the cells were re-suspended in PBS and irradiated as indicated above. A total of 50 × 10^3^cells/well was transferred to a black 96-well plate and the fluorescence intensity was measured in a fluorescence microplate reader (Synergy™ HT, Biotek Instruments) in triplicate wells (excitation: 498 nm, emission: 530 nm). Fluorescence intensity was normalized to the fluorescence values of non-irradiated cells.

### Cell cycle analysis and apoptosis

Cell cycle distribution of both adherent and sphere-forming cells was analyzed by flow cytometry at 48 h after treatment with chemotherapeutic agents and irradiation. Disaggregated cell suspensions were fixed with 75% ice-cold ethanol overnight and then incubated with 10 μg/ml propidium iodide (Sigma) in the presence of 500 μg/ml RNase (Sigma) for 60 min. Cell cycle data were collected on BD FACSCalibur Flow Cytometer (San Jose, CA) and analyzed using CellQuest data handling program. At least 10,000 events were acquired per experiment. The flow cytometer was calibrated with fluorescent standard microbeads (CaliBRITE Beads; BD Biosciences) for accurate instrument setting. Apoptosis was detected by nuclear Hoechst-33342 staining. After treatments, cells were permeabilized, fixed with ice-cold methanol-acetone (1:1), washed with PBS and then incubated with the DNA-specific dye Hoechst-33342 (Sigma, 5 μg/ml). The coverslips were mounted onto slides using Vectashield Medium and visualized under a fluorescence microscope (Zeiss LSM, 510 Meta) to examine the degree of nuclear fragmentation and chromatin condensation.

### Statistical analysis

Statistical analysis was performed with Statistical Package for the Social Sciences (SPSS) software (version 17; SPSS, Inc., Chicago, IL). Multiple comparisons between the three cells types were performed using the non-parametric Kruskal-Wallis test. The Mann-Whitney non-parametric test was applied to determine the difference between two groups. Level of significance was set at p < 0.05.

## Results

### Spherical colonies formation and self-renewal

**Figure 1:**
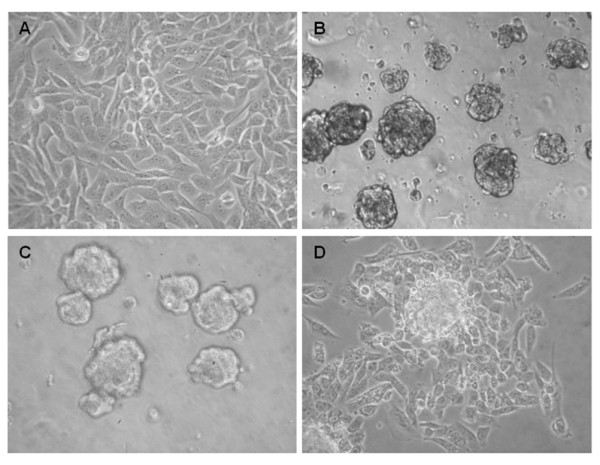
**Osteosarcoma cells form sarcospheres in serum-free medium and grow in an anchorage-independent manner**. (A) Monolayer culture of parental MNNG/HOS cell line. (B) Spherical colonies (sarcospheres) generated from single-cell suspensions of MNNG/HOS cells cultured in serum-free medium supplemented with growth factors in non-adherent conditions after 10 days. (C) Formation of secondary sarcospheres (7 days) derived from dissociated primary spheres. (D) Adherent expansion of a sarcosphere removed from the suspension culture and reintroduced into adherent conditions. (Original magnification: 200×).

To assess the presence of putative CSCs in osteosarcoma, MNNG/HOS cells (Figure 1A) were allowed to growth in serum-free medium in anchorage-independent conditions. After 2 days of culture, cells started to form floating spherical colony-like structures that continued to grow in size until they reached 50-100 μm in diameter (Figure 1B) at the end of the first week. The sphere-forming efficiency determined by the number of colonies formed per 60,000 total MNNG/HOS cells plated was of 5.3 ± 0.4% (n = 3). A second generation of sarcospheres was formed with an efficiency of 4.7 ± 0.7% (n = 3), yielding a stem cell frequency similar to that of the primary sphere formation assay (Figure 1C). This was further observed in a third round of sphere-forming assay, which demonstrates the ability of sarcospheres to self-renew under growth-constraining conditions. After plating in adherent conditions cells migrated from the colonies and started to adhere to the bottom of the flasks acquiring spindle-shaped morphology similar to the adherent MNNG/HOS cells (Figure 1D). Sarcospheres of the third generation, also termed as CSCs, were used in all subsequent experiments.

### Sarcospheres have attributes of mesenchymal stem cells markers and show trilineage differentiation potential

**Figure 2:**
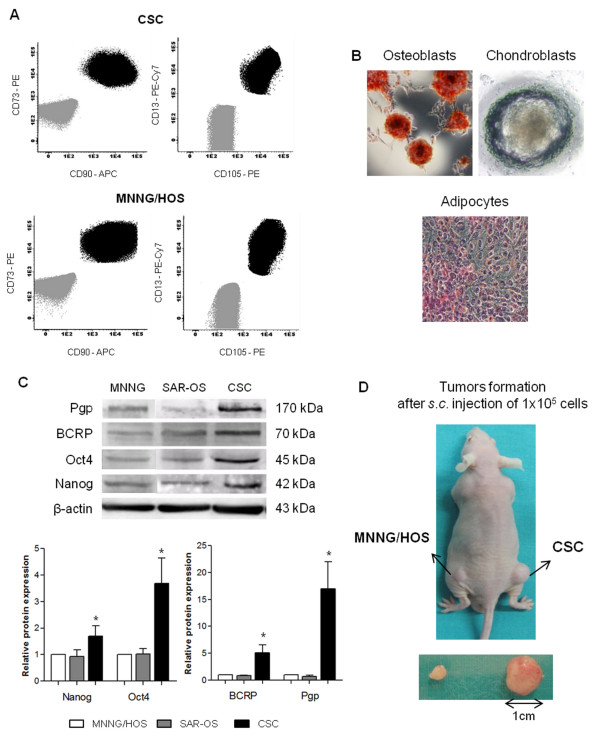
**Sarcospheres exhibit characteristics of MSCs, express stem-cell associated markers and are tumorigenic**. (A) Flow cytometry analysis of MSCs' surface markers in sarcospheres and MNNG/HOS cells. Both cells evidenced a strong positivity for CD73, CD90, CD13 and CD105. Unstained cells (grey) appear at the lower left quadrant as a negative fluorescence control. (B) Stainings demonstrating the differentiation potential of CSCs into mesenchymal lineages: osteoblasts (Alizarin Red), chondroblasts (Alcian Blue) and adipocytes (Oil Red), after incubation in osteogenic, chondrogenic or adipogenic inducing medium during 21, 16 and 14 days, respectively. (C) Western blot analysis of Oct4, Nanog, P-glycoprotein and BCRP in CSCs, SAR-OS and MNNG/HOS cells. β-actin was blotted as the loading control. The bottom panel shows the quantitative analysis of proteins (normalized to β-actin) expressed as a ratio of the levels found in MNNG/HOS cells, set as 1 for all proteins. The expression of all analyzed proteins is significantly enhanced in CSCs as compared with parental MNNG/HOS cells. The sphere-derived monolayer culture (SAR-OS) showed similar protein expression profiles to those observed in MNNG/HOS cells. Data represents mean ± standard error of the mean (SEM) of three independent experiments. *Significantly different from MNNG/HOS cells (p < 0.05). (D) Tumorigenic potential of CSCs. Balb/c nude mice were s.c injected with 1 × 10^5 ^of CSCs in the right flank and of MNNG/HOS in the left flank. Tumor growth was monitored every week. Representative image showed a CSC-derived tumor with a volume 7-fold higher to that induced by MNNG/HOS cells.

Because osteosarcoma originates from primitive mesenchymal bone-forming cells, third generation spheres were screened for the expression of cell surface proteins associated with MSCs by flow cytometry, according to the ISCT recommendations. The analysis revealed that sarcosphere-derived cells were positive for MSCs markers CD73, CD90, CD13 and CD105 (Figure 2A), and were negative for CD34, CD44, CD11b, CD19 and HLA-DR (data not shown). The lack of expression of these markers excludes hematopoietic progenitors and endothelial cells that are likely to be found in a MSC culture. There was no significant differential expression of these markers between sarcospheres and MNNG/HOS cells (Figure 2A).

The multipotency of isolated sarcospheres was evaluated through their ability to differentiate towards osteogenic, chondrogenic and adipogenic lineages upon culturing in specific differentiating conditions. Molecular markers of either osteogenic, chondrogenic or adipogenic commitment are shown in Figure 2B. Discrete foci of matrix mineralization were visualized by Alizarin Red S staining in cells cultured in osteogenic medium at 21 days. The differentiation in chondrogenic lineage was demonstrated by the intense Alcian Blue staining by day 16 under chondrogenic conditions. Visible Oil Red O-positive droplets containing-cells were seen at 14 days in adipogenic differentiating medium. Parental MNNG/HOS cells differentiated into osteoblasts, but were unable to differentiate towards adipocytes or chondrocytes (data not shown).

### Sarcospheres are enriched for stem cell specific transcription factors and drug efflux transporters

We investigated whether sarcospheres are enriched for the expression of the transcription factors Oct4 and Nanog, which are required for maintaining the pluripotency and self-renewal capacity of embryonic stem cells. Our western blot analysis revealed a 3.7-fold increase in Oct4 protein expression in sarcospheres compared to parental MNNG/HOS cells (Figure 2C). The expression levels of Nanog were modest, but significantly increased (1.7-fold) in sarcospheres compared with MNNG/HOS cells. Another characteristic of stem-like cells is the enhanced expression of the drug efflux transporters P-glycoprotein and BCRP that protects them from damages induced by cytotoxic agents. Comparative analysis revealed a significant (p < 0.05) 17-fold increased expression of Pgp and a 5.0-fold increase of BCRP in sarcospheres in relation to MNNG/HOS cells (Figure 2C). To further confirm if sarcospheres generate a differentiated progeny similar to the parental cells, we analyzed the expression levels of both stem cell markers Oct4 and Nanog and of ABC-related transporters in SAR-OS cells. As shown in Figure 2C, the four proteins that were upregulated in sarcospheres, returned to the expression levels found in parental MNNG/HOS cells, indicating that sarcospheres upon being cultured under differentiating conditions re-acquire the phenotype of differentiated parental cells.

### Sarcospheres have enhanced tumorigenic ability

Other characteristic that defines CSCs is their ability to initiate tumors *in vivo*. To verify if sarcospheres have enhanced tumorigenicity when compared with parental cells, equal numbers of cells (1 × 10^5^) derived from sarcospheres and of MNNG/HOS culture were injected subcutaneously in opposite flanks of nude mice (n = 3). Both cell fractions had the capacity to form tumors; however the size of the tumors derived from sarcospheres were significantly larger when compared with that from MNNG/HOS cells and started growing earlier. After 3 weeks the sarcosphere-derived tumors had an average volume of 158.3 ± 2.2 mm^3^, in contrast to the 22.8 ± 9.3 mm^3 ^of MNNG/HOS-derived tumors (Figure 2D). These results support the hypothesis that sarcospheres are enriched in stem-like cells with enhanced tumorigenic potential and that parental MNNG/HOS cells contain a small fraction of stem cells (able to generate tumors) but most of them are non-stem cells.

### Metabolic activity of sarcospheres during differentiation

**Figure 3:**
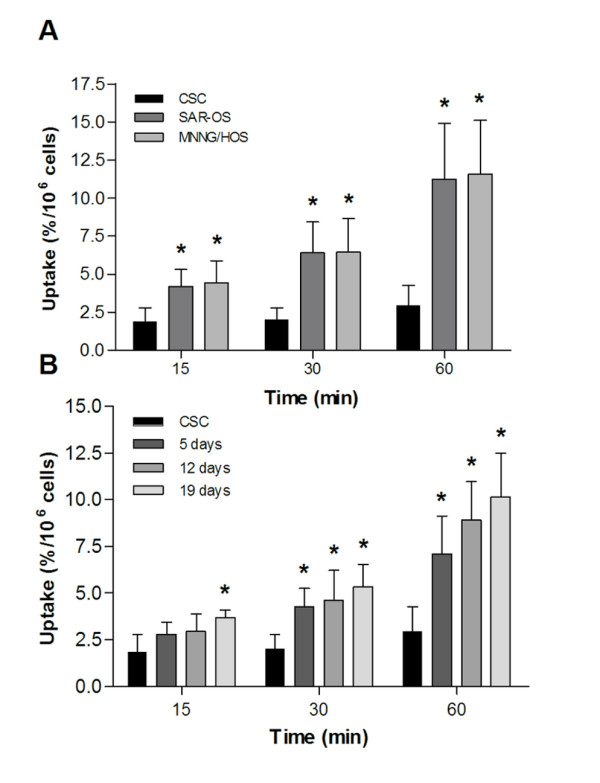
**FDG uptake of CSCs and of adherent cells**. Single cell suspensions were incubated with 0.75 MBq/mL of FDG at 37°C. At 15, 30 and 60 min, samples were collected and radioactivity of the pellet and supernatant was measured in a radioisotope calibrator well counter. Results are reported as the percentage of cell radioactivity associated with the total radioactivity added, normalized per million of cells. (A) Uptake of FDG in CSCs, SAR-OS and MNNG/HOS cells. At 60 min, the cellular uptake of FDG in CSCs was of 4-fold lower than that in both adherent MNNG/HOS and SAR-OS cells. (B) Uptake of FDG during differentiation of CSCs. FDG uptake increased progressively with the number of days of culture of CSCs in differentiating conditions, acquiring a metabolic profile similar to that of MNNG/HOS cells after 19 days. Data are shown as the mean ± standard deviation of four (n = 4, A) and three (n = 3, B) independent experiments performed in triplicate. *p < 0.05 as compared with CSCs at the corresponding time-points.

The metabolic activity of sarcospheres was estimated based on the cellular uptake of FDG and compared with that of their adherent counterparts. FDG uptake reflects both transport and phosphorylation of glucose by viable cells and is a well-established method for assessing the metabolic activity in tumor cells. A relatively low uptake of FDG was observed in sarcospheres as compared with that in MNNG/HOS and SAR-OS cells (Figure 3A, Table 1). At 60 minutes, the mean value of FDG uptake in sarcospheres (2.94 ± 1.33%) was about 4-fold lower than that in MNNG/HOS cells (11.57 ± 3.55%) and SAR-OS (11.27 ± 3.62%). This relatively lower accumulation of FDG in sarcospheres could be related with the quiescent status of these cells. The metabolic changes occurring under differentiation conditions were analyzed through the measurements of cellular FDG uptake at different periods of incubation in serum-containing medium under adherent conditions. Upon transference to adherent conditions, it was observed a progressive increase in the cellular uptake of FDG within the incubation period (Figure 3B, Table 2). After 19 days of culturing, cells accumulated FDG with a similar pattern to the observed in the parental MNNG/HOS cells.

**Table 1:** FDG uptake in CSCs, SAR-OS and MNNG/HOS cells

	Uptake (%/10^6^)		
**Time (min)**	**CSCs**	**SAR-OS**	**MNNG/HOS**

15	1.86 ± 0.94*	4.16 ± 1.14	4.42 ± 1.47

30	2.01 ± 0.79*	6.39 ± 2.03	6.45 ± 2.19

60	2.94 ± 1.33*	11.27 ± 3.62	11.57 ± 3.55

Results are expressed as the mean ± standard deviation of four independent experiments.

*p < 0.05 compared with SAR-OS and MNNG/HOS cells at the corresponding time-points.

**Table 2:** Uptake of FDG during differentiation of CSCs

	Uptake (%/10^6^)			
**Time (min)**	**CSCs**	**5 days**	**12 days**	**19 days**

15	1.86 ± 0.94	2.80 ± 0.64	2.95 ± 0.94*	3.68 ± 0.42*

30	2.01 ± 0.79	4.24 ± 1.00*	4.61 ± 1.62*	5.33 ± 1.21*

60	2.94 ± 1.33	7.10 ± 2.03*	8.93 ± 2.05*	10.14 ± 2.35*

Results are expressed as the mean ± standard deviation of three independent experiments.

*p < 0.05 compared with CSCs at the corresponding time-points.

### Increased survival of sarcospheres following chemo- and radiotherapy, and reversal of resistance to DOX

Sarcospheres and both adherent MNNG/HOS and SAR-OS cells were assayed for their sensitivity to chemotherapeutic agents and irradiation. Cell survival response following all treatments was measured using the MTT viability assay.

**Figure 4:**
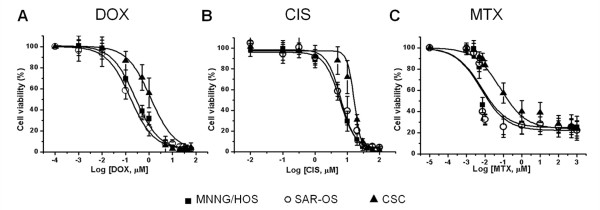
**Dose-response curves of CSC, SAR-OS and MNNG/HOS cells to DOX, CIS and MTX**. Cells were incubated with different doses of DOX (A), CIS (B) and MTX (C) during 48 hours. Cell survival was analyzed using the MTT assay. The mean IC_50 _values of all drugs in CSCs were significantly higher (p < 0.05) than those in SAR-OS and MNNG/HOS cells. Data are presented as the mean ± standard deviation of five independent experiments, performed in duplicate. The lines represent the fit to a sigmoid model.

Dose-response curves of sarcosphere and adherent cells treated with DOX, CIS and MTX are presented in Figure 4. All drugs inhibited cell viability in a dose-dependent manner, but sarcospheres were found to be more resistant as compared with their adherent counterparts. The mean IC_50 _values of all tested drugs in sarcospheres were significantly higher (Table 3) than those calculated in adherent cells. The differentiated progeny of sarcospheres (SAR-OS) displayed a drug sensitivity pattern similar to that of parental MNNG/HOS cells, as depicted in Figure 4 and Table 3, for the three drugs tested.

**Table 3:** IC_50 _values of chemotherapeutic drugs in CSCs, SAR-OS and MNNG/HOS cells

	IC_50 _(μM)		
	**DOX**	**CIS**	**MTX**

**CSCs**	0.80 ± 0.22*	14.71 ± 1.68*	0.050 ± 0.020*

**SAR-OS**	0.26 ± 0.04	7.96 ± 2.88	0.006 ± 0.001

**MNNG/HOS**	0.30 ± 0.07	6.61 ± 1.24	0.006 ± 0.001

Results are expressed as the mean ± standard deviation of five independent experiments performed in duplicate. *p < 0.05 compared to SAR-OS and MNNG/HOS cells *Abbreviations*: IC_50_, half maximal inhibitory concentration; DOX, doxorubicin; CIS, cisplatin; MTX, methotrexate. Cells were incubated with increasing concentrations of DOX (0-60 μM), CIS (0-80 μM) and MTX (0-1000 μM) during 48 hours. The cytotoxicity was evaluated using the MTT colorimetric assay and the IC_50 _were obtained from sigmoidal fitting of cell survival curves.

**Figure 5:**
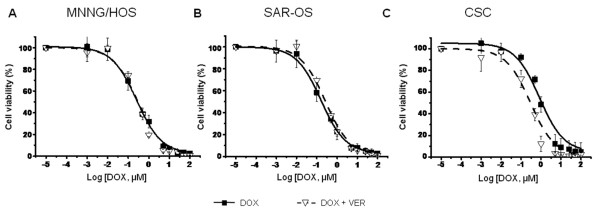
**Effects of VER on reversal of resistance to DOX in MNNG/HOS, sarcospheres and SAR-OS cells**. Dose response curves of DOX in the absence and in the presence of VER 10 μM. Data-points correspond to the mean ± standard deviation of at least three independent experiments performed in triplicate.

Reversal of resistance to DOX, by inhibition of Pgp and BCRP drug efflux pumps, was achieved using 10 μM of verapamil. Co-incubation with VER 10 μM, increased the sensitivity of sarcospheres to DOX and had no significant effects in adherent MNNG/HOS or SAR-OS cells, as illustrated in Figure 5. The IC_50 _of DOX in sarcospheres decreased significantly (p < 0.05) from 0.80 ± 0.22 μM to 0.29 ± 0.03 μM after treatment with VER, whereas in MNNG/HOS (IC_50 _= 0.27 ± 0.02 μM) or SAR-OS (IC_50 _= 0.24 ± 0.05 μM) the IC_50 _values did not differ significantly from those obtained without VER (MNNG/HOS: IC_50 _= 0.30 ± 0.07 μM; SAR-OS: IC_50 _= 0.26 ± 0.04 μM).

**Figure 6:**
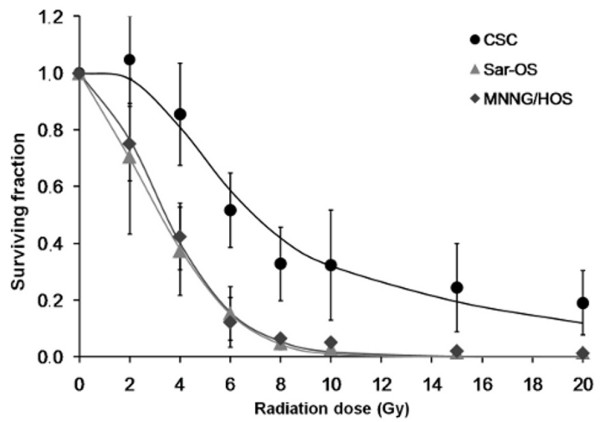
**Dose-response curves of CSC, SAR-OS and MNNG/HOS cells to ionizing radiation**. Exponentially growing cells were irradiated in a linear accelerator, at doses ranging between 2 and 20 Gy. Cell survival was analyzed after 7 days using the MTT assay. The LD_50 _for CSCs was significantly higher than that in SAR-OS and MNNG/HOS cells. Data are presented as the mean ± standard deviation of at least three independent experiments, performed in triplicate. The lines represent the fit to a linear-quadratic model.

Sarcospheres have also shown enhanced survival following irradiation, compared to adherent-growing cells. Cell survival curves and related parameters are presented in Figure 6 and Table 4, respectively. The surviving fraction of cells derived from sarcospheres was clearly superior when compared with those derived from adherent cells, in the wide range of tested doses. The irradiation survival curve of sarcospheres showed a shoulder in the initial portion of the curve (up to 2 Gy), and the corresponding mean lethal dose (LD_50_) was of 7.96 ± 3.00 Gy, significantly higher than the one observed in MNNG/HOS (3.36 ± 0.55 Gy, p < 0.05) and differentiated progeny SAR-OS (3.12 ± 1.38 Gy, p < 0.05) cells, and without any apparent shoulder on their survival curves. The α/β ratio, corresponding to the dose where cell killing from linear and quadratic components are equal, was of 18.6 Gy for sarcospheres, significantly higher than the one obtained for adherent cells.

**Table 4:** Cell survival parameters of CSCs, SAR-OS and MNNG/HOS cells after irradiation

Parameters	CSCs	SAR-OS	MNNG/HOS
LD_50 _(Gy)	7.96 ± 3.00*	3.12 ± 1.38	3.36 ± 0.55

α (Gy^-1^)	0.06411	0.10526	0.05421

β (Gy^-2^)	0.00353	0.03482	0.04323

α/β (Gy)	18.16*	3.02	1.25

R^2^	0.867 (n = 5)	0.999 (n = 3)	0.995 (n = 3)

Survival parameters were obtained from linear-quadratic model fitting of cell survival curves. The LD_50 _values correspond to the mean ± standard deviation of the indicated independent experiments, performed in triplicate. *p < 0.05 compared to SAR-OS and MNNG/HOS cells Abbreviations: LD_50 _- median lethal dose, α - probability of occurrence of a double-strand break induced by one ionizing particle, β - probability of two single-strand breaks combine and form a double-strand break, α/β - dose corresponding to the probability of occurrence of a double-strand break and the combination of two single-strand breaks be the same, R^2 ^- Adjusted R-squared.

### Radiation induces ROS production in adherent cells

**Figure 7:**
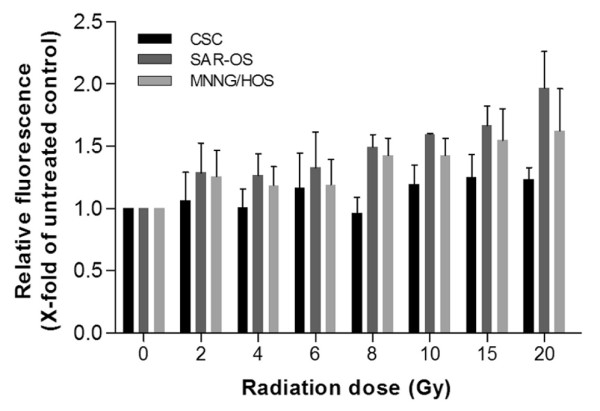
**Reactive oxygen species production in CSC, SAR-OS and MNNG/HOS cells following irradiation**. Single-cell suspensions were incubated with the fluorescent dye H_2_DCF-DA for 15 min in the dark. The levels of DCF fluorescence indicative of ROS generation were monitored within the first 60 min following irradiation (2-20 Gy) using a fluorescence microplate reader (excitation: 498 nm, emission: 530 nm) and normalized to the fluorescence values of sham-irradiated cells. Data represent normalized mean fluorescence intensity ± standard deviation, of three independent experiments performed in triplicate. Irradiation did not generate significant increases in ROS production in CSCs (p > 0.05) in relation to sham-irradiated cells. For adherent cells significant increases (p < 0.05) were observed for doses above 8 Gy.

The intracellular levels of ROS induced by irradiation were measured using the fluorescent dye H_2_DCF-DA. The measurements were performed within the first 60 min following irradiation and normalized to the controls. Both monolayer cultures (MNNG/HOS and SAR-OS) showed a dose-dependent formation of ROS (Figure 7). In these cells the increase observed in ROS production in relation to the non-irradiated cells was significant, even for the lower irradiation dose used (2 Gy). In opposite, irradiation did not induce a significant increase in ROS production in sarcosphere-derived cells. These results suggest that sarcospheres might have higher intrinsic antioxidant capacity than their differentiated progeny (SAR-OS) and MNNG/HOS cells, which may contribute for their higher radioresistance.

### Cell cycle progression following chemotherapy and radiation exposure and induction of apoptosis

**Figure 8:**
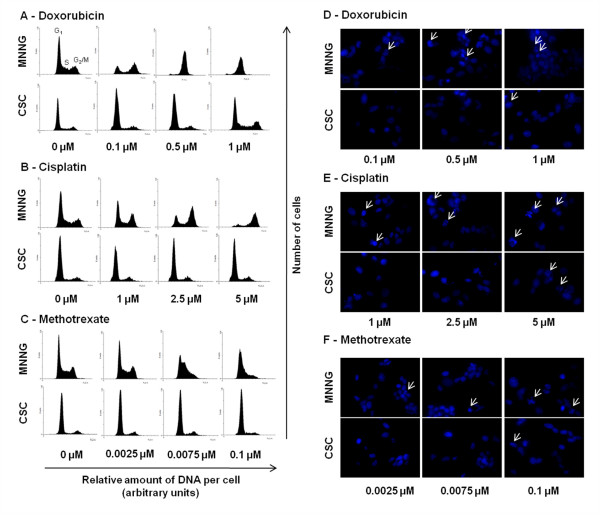
**Cell cycle and apoptotic responses of MNNG/HOS and CSCs to chemotherapeutic agents**. Representative cell cycle phase distribution and Hoescht-33342 nuclear staining of MNNG/HOS and CSCs after treatment with doxorubicin (A, D), cisplatin (B, E) and methotrexate (C, F). Cells were incubated with drugs at the indicated concentrations. After 48 h, cells were fixed and stained with propidium iodide for cell cycle analysis by FACS and with the DNA Hoescht-33342 dye to detect apoptosis. All drugs induced a cell cycle arrest and chromatin condensation in MNNG/HOS cells. In contrast, there were no significant changes in cell cycle distribution of CSCs and a few apoptotic cells were detected for the high drug concentrations. Fluorescence images were acquired using a fluorescence microscope with an original Magnification of × 630.

Analysis of cell cycle distribution was performed after 48 h of treating sarcospheres and adherent cells with chemotherapeutic drugs and ionizing radiation, using PI staining and flow cytometry. DOX and CIS, at doses near the IC_50 _values, caused a G2/M cell cycle arrest in a concentration-dependent manner in parental cells that was accompanied by a proportional decrease in the percentage of cells in the G1 phase (Figure 8A, B). The proportion of cells in the S phase was not significantly altered by any of the drugs. The anti-metabolite MTX that inhibits the synthesis of DNA, induced an arrest in the MNNG/HOS cell population in the G1/S phase and decreased the percentage of cells in the G2/M phase (Figure 8C). Similar effects were observed in cell cycle progression of SAR-OS cells (data not shown). In opposite, none of the drugs altered significantly the cell cycle distribution in sarcosphere-derived cells. To determine whether chemotherapeutic agents induced apoptosis, nuclei were stained with the Hoechst-33342 dye. The results obtained showed that all drugs induced an increase in chromatin condensation and formation of apoptotic bodies in both MNNG/HOS and SAR-OS adherent cells, for the tested doses. For sarcosphere-derived cells, the morphological changes typical of apoptosis were less evident and only became visible at higher dosages (Figure 8D-F).

**Figure 9:**
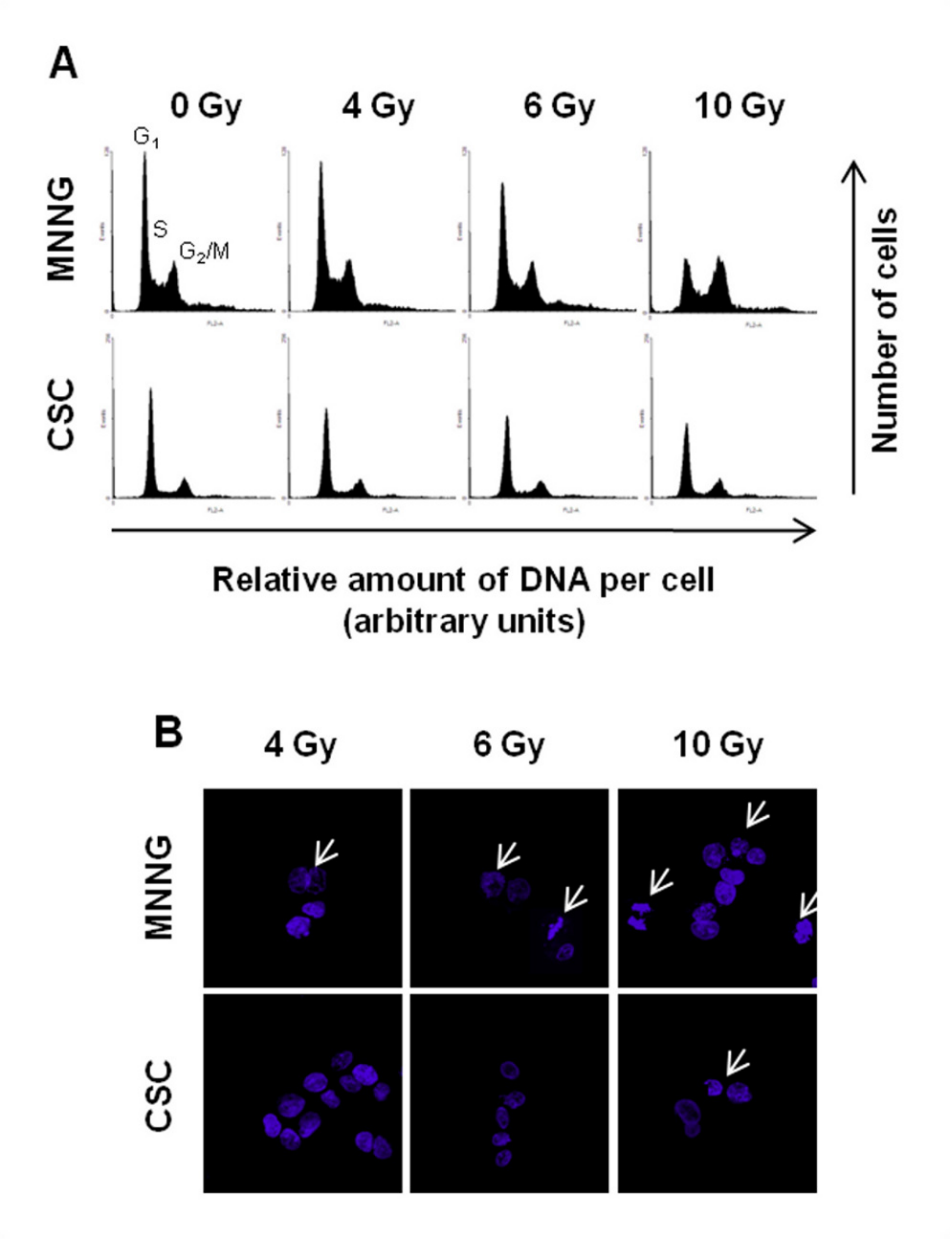
**Cell cycle and apoptotic responses of MNNG/HOS and CSCs to irradiation**. Representative cell cycle phase distribution (A) and Hoescht-33342 nuclear staining (B) of MNNG/HOS and CSCs after irradiation at indicated doses. Forty-eight hours after irradiation, cells were fixed and stained with propidium iodide (PI) for cell cycle analysis by flow cytometry and with the DNA Hoescht-33342 dye to detect apoptosis. Irradiation induced a dose-dependent cell cycle arrest at G2/M phase and apoptosis in MNNG/HOS cells and had no significant effects on cell cycle progression in CSCs with minimal induction of apoptosis. Fluorescence images were acquired using a fluorescence microscope with an original magnification of × 630.

Cell cycle analysis of irradiated adherent cells showed a dose-dependent cell cycle-arrest at G2/M phase that was accompanied by a proportional decrease of cells in G1 phase. As observed with chemotherapeutic drugs, irradiation has not induced significant changes in the cell cycle phase distribution of sarcosphere-derived cells compared with sham-irradiated controls (Figure 9A). Micrographs of Hoechst-33342 staining showed the formation of apoptotic membrane blebbing and chromatin condensation in MNNG/HOS and SAR-OS cells irradiated at doses of 4, 6 and 10 Gy. Focuses of chromatin condensation in sarcospheres were only visualized with extreme doses of irradiation (Figure 9B).

## Discussion

In this study we aimed to identify the presence of putative CSCs in a human osteosarcoma cell line and to evaluate their role in resistance to chemo- and radiotherapy. Our findings provide evidence that this osteosarcoma cell line contains a subpopulation of cells with stem-like properties as demonstrated by the formation of spherical clones in serum-free medium under anchorage-independent conditions. These results are in accordance with those previous reported by Gibbs *et al. *that demonstrated the presence of stem-like cells in distinct bone sarcomas when cultured in stressful growth conditions [16]. This method has been widely used in the prospective isolation of cells with stem-like properties from several malignancies, and is particularly useful when specific markers have not been identified, as is the case of CSCs for most tumors [20-23].

The isolated cells were found to express MSC's surface markers CD73, CD90 and CD13 and were able to differentiate into the osteoblastic, chondrocytic and adipocytic lineages under standard culture differentiating conditions, which indicates that they remain mesenchymal and preserve some degree of the MSC's plasticity. However, the expression pattern of MSC-related antigens cannot be used as a specific marker of stem-like cells in osteosarcoma, since parental MNNG/HOS cells exhibited an immunophenotype similar to that of sarcospheres. This is in line with the theory that osteosarcoma originates from a primitive MSC in a consequence of impaired differentiation into osteoblasts that undergo malignant transformation. Therefore it is conceivable that more differentiated parental MNNG/HOS cells retain some properties of MSC, including the expression of cell surface markers and that the overlap of immunophenotype markers is related with the stage of differentiation of MSCs at the time of the mutation [24]. Moreover, after culturing in specific differentiation conditions, MNNG/HOS cells differentiated towards osteoblasts, which indicate these cells still have some propensity of the original lineage. In addition, we found that cells derived from sarcospheres expressed high levels of Oct4 and Nanog (Figure 2C), which are key transcription factors required for the maintenance of self-renewal and pluripotency of embryonic stem cells (ES). These attributes of ES cells have regularly been identified in subsets of stem-like cells derived from mesenchymal tumors and of many other solid tumors, and appear to be critical to the phenotype of tumor-initiating cells [25-27].

Two other fundamental properties of cancer stem-like cells are their ability to self-renew and to generate differentiated progeny. The sphere-forming capacity of these cells that was observed following three serial passages under selective culture conditions demonstrated the presence of a self-renewing cell population within the MNNG/HOS cell line. Furthermore, when transferred to adherent plates and allowed to grow in serum-containing medium, sarcospheres were able to expand in monolayer acquiring the morphological features and biological behavior of parental MNNG/HOS cells. We noticed that after 19 days in differentiating culture conditions, sarcosphere-derived cells (SAR-OS cells) started to proliferate with the same doubling-time as the MNNG/HOS cells around 24 h (data not shown), displayed similar glucose uptake as assessed with FDG and showed a significant decrease in the expression levels of the proteins associated with a stem cell phenotype (Oct4 and Nanog) and of the ABC-related transporters (Pgp and BCRP), reaching the levels of parental MNNG/HOS cells. The degree of FDG accumulation is considered a good indicator of the metabolic status of tumor cells. This fluorinated glucose analogue is widely used for detecting and staging of malignant tumors based on the enhanced glucose utilization of tumor cells when compared to non-tumoral tissues [28,29]. This increased accumulation of FDG is related to a change in the metabolism of tumor cells that switches from oxidative phosphorylation to glycolysis, even in the presence of high levels of oxygen. This process, the so-called Warburg effect, results in a much less efficient mechanism for energy generation, and thus an increase in the requirement for glucose uptake, but is important in providing building blocks to support cancer cell proliferation [30,31].

We found that sphere-forming cells, in comparison with the parental MNNG/HOS cells, accumulated significantly lower amount of FDG (Figure 3A). Moreover, after being placed in differentiation culture conditions, we observed a marked and progressive increase in the cellular uptake of FDG until reaching the values of MNNG/HOS cells after 19 days (Figure 3B). This progressive increase in cellular FDG uptake indicates that there are dynamic changes in glucose metabolism occurring during the differentiation process and that undifferentiated cells, as stem like-cells, are likely to have low energy requirements. This could be related with the fact that this fraction of cells is entering into a quiescent status and divides infrequently. This is in line with previous studies suggesting that quiescent cells reduce their glucose uptake and metabolic rate in contrast with highly proliferative cells [32].

Some studies have shown that stem cells can become quiescent without losing their proliferative potential [14]. This has been referred as an intrinsic defense mechanism of CSCs that they use against chemotherapeutic drugs targeting rapidly dividing cells [33]. As a result, at least some of the stem-like cells are able to survive and be responsible for tumor regrowth after therapy. The lower FDG accumulation by stem-like cells can have important clinical implications, as PET imaging with FDG is commonly used for monitoring tumor response to therapy, by measuring changes in FDG uptake. In general, tumors with low accumulation of FDG after therapy are considered to be a good prognostic factor. Based on our observations, this reading can be biased by the fact that surviving stem-like cells might be few in number and might not accumulate FDG efficiently due to their quiescence, a status that can be maintained for a defined period before they return to a proliferative state and initiate tumor recurrence.

The enhanced tumorigenic ability of sarcospheres was demonstrated *in vivo *in immunocompromised mice. The animals injected with 100,000 sphere-derived cells developed massive tumors with approximately 150 mm^3 ^of volume at 4 weeks, whereas the injection of the same number of MNNG/HOS cells induced tumors with a 7-fold lower volume (approximately 20 mm^3^). Based on previous studies, the injection of few stem-like cells derived from mesenchymal neoplasms can be responsible to initiate a tumor due to their enhanced capacity for self-renewal and a more plastic capacity that enables them to adapt to the stringent environment of the xenografts [30,34]. It seems reasonable that the tumors arising from the MNNG/HOS cells results from the presence of a subset of stem-like cells that are sufficient to initiate tumor formation.

The chemo- and radiosensitivity assays clearly demonstrated the higher resistant profile of spherical clones when compared with parental MNNG/HOS cells. The mean IC_50 _values of all tested drugs (DOX, CIS and MTX) in sarcospheres were significantly higher (up to three to four-fold) than those obtained in adherent MNNG/HOS and SAR-OS cells (Table 3). One of the mechanisms that have been proposed to explain the chemoresistance of CSCs is the activity of certain ABC transporters that mediate drug efflux, preventing the intracellular accumulation of chemotherapeutic agents at toxic levels. The up-regulation of these transporters has been observed in CSCs of several malignancies and is also the basis for the Hoechst-33324 dye exclusion assay to isolate a side-population (SP) enriched with cancer stem-like cells in cell lines and tumor samples [35]. Our data demonstrated a significant high expression of Pgp and BCRP in spherical colonies compared with parental cells (Figure 2C), which might explain the higher resistance of CSCs to DOX and MTX, since these drugs are substrates of those transporters [12,36]. The reversal of resistance to DOX that was observed in sarcospheres after co-incubation with VER sustains this hypothesis. It has long been known that VER restores drug accumulation sensitizing resistant tumor cells through inhibition of drug efflux pumps [37]. VER is a non-specific inhibitor of ABC transporters and is itself a transport substrate of Pgp and BCRP competing with drugs for the transporter, blocking the efflux of the chemotherapeutic agent. Therefore is seems reasonable that both proteins are functionally active and contribute for the drug resistance phenotype of CSCs, at least to DOX which is the main chemotherapeutic drug used in the treatment of osteosarcoma.

In addition to an increased capacity for drug efflux, other mechanisms may co-exist, like alterations in cell cycle, enhanced DNA repair capacity, reduced apoptosis and expression of specific drug-detoxifying enzymes [38]. High activity of the detoxification enzyme aldehyde dehydrogenase (ALDH1) has been found in osteosarcoma CSCs as well as in other solid tumors, and is referred as a possible drug resistance mechanism for both normal and malignant stem cells [39,40]. This enzyme is responsible for the oxidation of intracellular aldehydes, thereby mediating self-protection and resistance to some alkylating agents (e.g. cisplatin) used in cancer treatments [41].

The ability of stem-like cells to enter in a quiescent or slow-dividing state can also contribute for their resistance to conventional therapies that target proliferating cells. The low energy requirement evidenced by the small accumulation of FDG in sarcospheres is consistent with a slow dividing rate of these cells. Likewise, the mean LD_50 _values obtained from irradiation cell-survival curves were significantly superior for sarcospheres as compared with adherent cells (Table 4). The survival curves of sarcospheres clearly showed a shoulder at lower and, therefore, clinically relevant doses of irradiation, which is probably due to an enhanced capacity to repair potential lethal damages. In contrast, no obvious shoulder was observed in both adherent MNNG/HOS and SAR-OS cells. This is consistent with the higher α/β ratio that was obtained for sarcospheres (18.16 Gy) compared to that of the MNNG/HOS cells (1.25 Gy). In general, cells displaying high α/β ratios are more resistant to cell dead induced by lethal DSB and have an enhanced capacity of DNA repair [42]. It is well established that cell killing after exposure to ionizing radiation is partially mediated by free radicals. Consistent with the increased radioresistance, were the decreased production of ROS levels in sarcosphere-derived cells, compared with adherent counterparts (Figure 7), potentially as a result of increased levels of free radical scavengers (e.g., glutathione and superoxide dismutases). These results are in agreement with previous data reported by others in cancer-initiating cells of brain tumors [43] and breast cancer [44,45]. They found that stem cell-enriched subpopulations contained low levels of ROS and developed less DNA damages compared with non-stem counterparts, and that those low levels of ROS were associated with increased expression of free radical scavenging systems. Notably, the depletion of ROS scavengers, via pharmacological depletion of glutathione, increased the radiosensitivity of breast CSCs, which demonstrated the importance of anti-oxidative defenses in radioresistance and survival of stem-like cells. The general low ROS concentration found in normal tissue stem cells, compared with their cellular descendants suggests that stem cells have conserved this attribute for protecting their genome from endogenous and exogenous ROS-mediated damage [45].

The absence of significant alterations in cell cycle progression of sarcospheres following irradiation and drug exposure as well, suggests that they possess highly activated basal DNA repair mechanisms and possibly enhanced efficiency on DNA damage response activity that restrain them from undergoing apoptosis. In fact, we observed that sarcospheres are less susceptible to apoptosis as compared with parental MNNG/HOS cells, since the typical signs of apoptosis were only visible for higher doses of irradiation and drugs. Both treatments induced apoptosis and a G2/M cell cycle arrest in a dose-dependent manner in adherent cells indicating a cellular response to DNA-induced damages.

Our results are in line with previous findings observed in CSCs isolated from mesenchymal neoplasms showing increased chemoresistance with respect to their adherent counterparts [46-48]. Such limited effectiveness of standard therapies suggests that they possess innate resistance mechanisms allowing them to survive and initiate tumor recurrence. The high levels of Pgp/BCRP expression and the relative quiescence observed in sarcospheres compared with the bulk population come out as potential resistance mechanisms operating in osteosarcoma stem-like cells. Nevertheless we cannot exclude other mechanisms such as the up-regulation of anti-apoptotic and down-regulation of pro-apoptotic pathways, as well as active DNA repair that can contribute to the overall resistance of CSCs to standard therapies.

## Conclusions

Our study provides strong evidence that MNNG/HOS osteosarcoma cell line is enriched in CSCs with enhanced tumorigenic potential and increased resistance to conventional therapies. The relatively higher resistance of CSCs to the main chemotherapeutic agents recommended by EURAMOS-1 might contribute to the currently static survival rate observed in osteosarcoma patients. Further studies in CSCs derived from patients' specimens are needed for a continued clarification of the role of these cells in therapy response and therefore to contribute for the establishment of novel therapeutic strategies.

## Competing interests

The authors declare that they have no competing interests.

## Authors' contributions

SMN performed the irradiation studies and ROS detection, western blot analysis and FDG uptake, participated in data analysis and in the preparation of the manuscript. AOG carried the chemosensitivity assays and participated in the animal studies; AAC performed cell cycle and apoptotic experiments; AAP performed the flow cytometry analysis of stem cell markers, PSC supervised the irradiation experiments, AJA participated in the design of study and supervised the studies with radiotracers; CMG performed the study design, supervised the experimental work and the data analysis and prepared the manuscript. All authors read and approved the final manuscript.

## Pre-publication history

The pre-publication history for this paper can be accessed here:

http://www.biomedcentral.com/1471-2407/12/139/prepub

## Supplementary Material

Additional file 1 **Ethics Committee Approval**. Ethics committee approval by the Faculty of Medicine of the University of Coimbra. (File is in Portuguese). Click here for file
