# Supplementary material for: Therapeutic implications of an enriched cancer stem-like cell population in a human osteosarcoma cell line
Source: BMC Cancer. 2012 Apr 4;12:139. doi: 10.1186/1471-2407-12-139 (PMC3351999; doi:10.1186/1471-2407-12-139)
Supplement: Additional file 1 — Ethics Committee Approval. Ethics committee approval by the Faculty of Medicine of the University of Coimbra. (File is in Portuguese). [file 1471-2407-12-139-S1.PDF]

COMISSÃO DE ÉTICA DA FMUC

Of. Refª **38-CE-2011**

Data 30/ 06 /**2011**

c/c à Exma. Senhora Doutora Célia Maria Freitas Gomes

Exmo Senhor

Prof. Doutor Manuel Santos Rosa

Director da Faculdade de Medicina de

Universidade de Coimbra

Assunto: Pedido de parecer à Comissão de Ética - Projecto de Investigação autónomo.

Investigador(a) Principal: Célia Maria Freitas Gomes

Titulo do Projecto: *"Células cancerígenas estaminais e resistêncua à quimioterapia no osteossarcoma"*.

A Comissão de Ética da Faculdade de Medicina, após análise do projecto de investigação supra identificado, decidiu emitir o parecer que a seguir se transcreve:

A Comissão de Ética da Faculdade de Medicina, após análise do projecto de investigação supra identificado, decidiu emitir o parecer que a seguir se transcreve: ***"Parecer Favorável".***

Queira aceitar os meus melhores cumprimentos.

O Presidente,

Prof. Doutor João Manuel Pedroso de Lima

GC
